# Our Journal

**Published:** 1884-03

**Authors:** 


### Editorial.

### OUR JOURNAL.

Again THE ATLANTA MEDICAL AND SURGICAL JOURNAL unfurls her banner to the breeze, under her true name and colors, and thus commences her third series, under auspices never before so flattering, or prospects more bright. In thus entering again the race with our sister publications—the race for the highest plane to be reached by a first-class medical journal—we do so with love and good fellowship for all our competitors, without enemies to punish, or friends to reward. With our old and tried motto, "*Pax et scientia sed veritas sine timore*," ever in sight, we hope to be able to steer our little bark clear of all troubled and muddy waters—only engaging in conflicts, when we feel sure that such a course will be in the interest of science and progression.

With such feelings and desires, which we pray will ever actuate us in conducting this journal, we hope to steer clear of all personal issues, in which the profession have no interest, but to devote our space to the advance of science, and in the interest of our readers.

In commencing the new series, we have thought it not inappropriate, to give in this connection an outline of the history of THE JOURNAL, from its commencement up to date. The first number of THE ATLANTA MEDICAL AND SURGICAL JOURNAL was issued September 1st, 1855, from the press of the long-since suspended, Atlanta *Intelligencer*, and was brought into existence under the editorial management of Drs. J. P. Logan and W. F. Westmoreland, and was conducted by those gentlemen consecutively until September 1st, 1860. At this date, Dr. J. G. Westmoreland assumed the editorial management, and issued it regularly until the summer of 1861, when THE JOURNAL was suspended on account of the then commencing hostilities, in the great conflict between the North and the South.

From its suspension in the summer of 1861 to ~~the fall of 1867~~, there was no issue of THE JOURNAL; at the latter date the publication was commenced again under the editorial management of Drs. J. G. and W. F. Westmoreland, and continued ~~one year~~. It was found at the expiration of that time that the people, and particularly the doctors of the South, were so impoverished by the war that the publication, if continued, would be at a great financial sacrifice to the publishers, and it was again suspended, to be re-issued in ~~March~~, 1871, under the same editorial management, and continued up to 1874.

From the latter date it was regularly issued, under the editorial management of Drs. Robert Battey, W. A. Love and H. V. Taliaferro for two years and a half, when Drs. W. F. Westmoreland and W. S. Kendrick became its editors, and issued it continuously until the fall of 1878, when Drs. J. G. and R. W. Westmoreland assumed the editorial management and conducted it for two years.

In 1880, Drs. J. B. Baird and J. T. Johnson became the editors of THE JOURNAL. For reasons unnecessary to discuss here, the name was changed to *The Atlanta Medical Register*, with ATLANTA MEDICAL AND SURGICAL JOURNAL in small letters, and in brackets just beneath. THE JUORNAL, under this name, was edited by them until the summer or fall of 1883, when Dr. J. H. Logan became its editor, and remained in charge until it was purchased by Jas. P. Harrison & Co. a few weeks ago.

It will be seen by this short sketch, that THE ATLANTA MEDICAL AND SURGICAL JOURNAL has had, in the past twenty-nine years, quite a number of editors and many changes in the editorial department, but never, until the past few years, when under the name of *Atlanta Medical Register*, has there been a change in the editorial department without the most perfect understanding, all having been amicably and satisfactorily arranged, both with the retiring and incoming editors. Never an unpleasant feeling, or jar, or the least friction, preceding, during, or following a change of editors. All was done in harmony, by mutual agreement and without feeling.

We feel, that we can predict the same harmony for the present editorial staff—and without hesitancy pledge our readers that we shall leave nothing undone to make this a first-class Medical Journal. In fact, we shall not be satisfied with less.

In commencing the publication of a Medical Journal, as we have this, without time to become familiar with the exchanges at hand, and without being abreast with the current medical topics and medical literature of the day—hurried to press to meet a specific promise, we fear that we will not, in this our first number, reach that standard which we are determined ultimately to attain.

As to our publishers and the mechanical department of THE JOURNAL, it is unnecessary for us to say more than simply to announce, that it is to be issued from the great publishing house of James P. Harrison & Co., which is not equaled by any in the State, or excelled by any in the South, in its facilities to publish, not only a medical journal, but any character of publication that may be presented. As will be seen from the prospectus, they offer the most liberal terms to contributors, as the following published proposition will demonstrate: "A special feature of THE JOURNAL will be the illustration of all matter that requires illustrating. These illustrations will be furnished by the publishers free of charge to contributors." What medical publication does as much? Such a liberal proposition from the publishers can but attract first-class contributors, and will greatly assist us in making this JOURNAL what we desire it to be.

